# Re-Emergence of the Great Mimicker: Congenital Syphilis in a Low Prevalence Setting

**DOI:** 10.3390/idr18050107

**Published:** 2026-09-17

**Authors:** Rabab AlGhaithi, Shawg AlSalamah

**Affiliations:** 1 Pediatric Infectious Disease, King Fahad Medical City, Riyadh 11525, Saudi Arabia; 2 General Pediatrics, King Fahad Medical City, Riyadh 11525, Saudi Arabia; shoug197@hotmail.com

**Keywords:** congenital syphilis, syphilis, congenital infection, mother to child transmission, pre-natal infection

## Abstract

Background: Congenital syphilis remains a significant cause of adverse birth outcomes despite effective screening and treatment. Data on its epidemiology in Saudi Arabia are limited. This study aims to describe the incidence, clinical characteristics, and outcomes of congenital syphilis at a tertiary pediatric center in Riyadh, Saudi Arabia, and to identify gaps in antenatal care and prevention. Methods: We conducted a retrospective chart review of all infants diagnosed with congenital syphilis (aged 0–24 months) and pregnant women with positive syphilis serology who presented to our center between January 2021 and December 2025. Cases were classified as highly probable, possible, less likely, or unlikely based on Center for Disease Control (CDC) and European criteria. Incidence rates were calculated per 1000 live births. Results: Over the five-year study period, there were 11,722 live births and 12 cases of probable or possible congenital syphilis, yielding an incidence rate of 1 per 1000 live births. An additional two cases were classified as less likely due to successful prevention of mother-to-child transmission. The majority of cases (*n* = 7) were attributed to inadequate maternal treatment. Three cases of abortion and intrauterine fetal demise were identified, probably due to untreated maternal syphilis. Most infants were asymptomatic at birth; however, one patient presented with hepatosplenomegaly, cytopenia, and cholestasis, while two had abnormal long bone findings. No cases of neurosyphilis were identified. Five infants were born to mothers with suspected false-positive serology but were managed as possible cases due to incomplete follow-up. Favorable neurodevelopmental outcomes were observed in all patients who completed follow-up (*n* = 5). Conclusions: We report congenital syphilis incidence rates that align with the global upward trend. We also identified multiple missed opportunities for screening and early treatment—findings that can inform future guidelines and enhance awareness among healthcare providers and the public.

## 1. Introduction

Syphilis is a sexually transmitted infection (STI) caused by the spirochete *Treponema pallidum*. In the pediatric population, it is typically acquired from untreated or inadequately treated maternal infection during the primary, secondary, or latent stage. The spirochete crosses the placenta, and once congenital syphilis develops, multiple serious outcomes can occur in up to 20% of the cases, including multisystem complications, abortion, stillbirth, and intrauterine fetal demise [1,2]. Without treatment, the rates of transmission from the mother to the fetus are high, reaching up to 100% in primary and secondary syphilis, and 40%, and 10% in early latent and latent syphilis, respectively [3]. Prevention of mother-to-child transmission (MTCT) of syphilis can be achieved with early and appropriate treatment once the diagnosis is made during pregnancy; with rates of transmission dropping to 1%, 18%, 41% in the first, second and third trimesters [3,4]. Therefore, screening for syphilis during pregnancy is recommended by the World Health Organization (WHO) and is implemented in many settings globally, including our region [5,6].

Despite effective screening programs and the availability of cost-effective treatment, congenital syphilis has been increasing in many areas in recent years. The WHO estimated the global burden of congenital syphilis in 2022 to be approximately 700,000 cases, with 390,000 associated adverse birth outcomes. These outcomes included an estimated 150,000 early fetal deaths and stillbirths, 70,000 neonatal deaths, 55,000 preterm or low-birthweight newborns, and 115,000 infants with a clinical diagnosis of congenital syphilis [5].

Numerous sources describe a growing trend in maternal and congenital syphilis since the early 2010s, with particularly marked increases in some high-income countries, such as the United States. For example, between 2020 and 2021, the incidence of primary and secondary syphilis among US females aged 15–44 years increased by 52.3%, and the incidence of congenital syphilis increased by 30.5% compared to 2020, representing a 219.3% increase relative to 2017 [7]. The re-emergence of syphilis in adults is attributed to multiple risk factors, including unprotected sex, homosexuality, having multiple sexual partners, unmarried status, and other high-risk behaviors (e.g., intravenous drug use and previous STIs) [8,9].

A similar increase has been observed in the Middle East region in recent years. Maternal syphilis prevalence in the region increased from 0.69% in 2012 to 0.77% in 2016 [10]. Published data regarding congenital syphilis and syphilis in pregnancy from the Middle East remain extremely limited, despite the region accounting for 17.1% of the estimated global burden of congenital syphilis [11].

Adherence to screening guidelines has shown significant impact in neighboring countries such as Oman, which was certified by WHO in 2022 for eliminating mother-to-child transmission of HIV and syphilis, becoming the first country to achieve this in the Eastern Mediterranean Region [12].

In this paper, we aim to address the gap in knowledge regarding the local epidemiology of congenital syphilis and to identify areas for improvement in antenatal care and prevention.

## 2. Methods

This retrospective study involves a review of patient charts who are diagnosed with congenital syphilis (between the ages 0–24 months) or managed for mother-to-child transmission of syphilis during the period from January 2021 until December 2025. Also, we included all pregnant women who presented to our center and had a positive syphilis serology screening and confirmatory result. At our institution, antenatal syphilis screening is performed for every pregnant woman during the first trimester, or at any trimester upon her initial presentation to care. A chart review of the included mothers and their respective infants was done. Serology testing is done by following the reverse screening algorithm. The initial serum screening test is done via chemiluminescent microparticle immunoassay (CMIA) to detect immunoglobulin G (IgG) and immunoglobulin M (IgM) antibodies against *Treponema pallidum*. If the result is weakly reactive or reactive, further confirmatory testing is done via non-treponemal test rapid plasma reagin (RPR) and treponemal test treponemal pallidum hemagglutination assay (TPHA).

To increase the sensitivity of detection, we included mothers with an untreated syphilis infection—diagnosed based on serology and seen by an infectious disease specialist- and who have history of recurrent abortions without a clear medical explanation, a stillborn or intrauterine fetal demise (IUFD).

Incidence rates of congenital syphilis were calculated by dividing the cases of proven, probable and possible congenital syphilis over the annual numbers of total live births in our center.

The available data such as age, gender, clinical presentation (growth parameters; vital signs; end organ involvement), complete blood counts (CBC), liver function tests (LFT); imaging, treatment history (type of antibiotic, duration of treatment), and results of serological tests were recorded.

Congenital syphilis is defined as highly probable, possible and less likely based on the Center of Disease and Control and European criteria [13,14] as the following:

- Confirmed or highly probable case:
  ○ Any neonate with abnormal physical examination consistent with congenital syphilis
  ○ Any neonate with non-treponemal test 4 folds or higher than the mother’s at birth or a positive darkfield test or PCR of placenta, cord, lesions, or body fluids or a positive silver stain of the placenta or cord, which is not available in our Facility.
- Possible case:
  ○ Any neonate with normal physical examination and a serum quantitative nontreponemal serologic titre equal to or less than fourfold of the maternal titre at delivery
  ○ The mother was not treated, was inadequately treated, or has no documentation of having received treatment.
  ○ The mother was treated with erythromycin or a regimen other than those recommended.
  ○ The mother received the recommended regimen, but treatment was initiated < 30 days before delivery.
- Less likely case: Any neonate who has a normal physical examination and a serum quantitative nontreponemal serologic titre equal or less than fourfold of the maternal titre at delivery and both of the following are true:
  ○ The mother was treated during pregnancy, treatment was appropriate for the infection stage, and the treatment regimen was initiated ≥ 30 days before delivery.
  ○ The mother has no evidence of reinfection or relapse.
- Unlikely: Any neonate who has a normal physical examination and a serum quantitative nontreponemal serologic titre equal to or less than fourfold of the maternal titre at delivery, and both of the following are true:
  ○ The mother’s treatment was adequate before pregnancy.
  ○ The mother’s nontreponemal serologic titre remained low and stable before and during pregnancy and at delivery (RPR ≤ 1:4).

Inadequate maternal treatment is defined as any failure to adhere to CDC guidelines, whether in regimen, dosage, or timing.

## 3. Results

Over the five-year study period, there were 11,722 live births and 12 cases of probable or possible congenital syphilis. During the study period, our institution recorded a total of 22,913 pregnant women, and 9188 infants. We identified two cases with a less likely diagnosis of congenital syphilis due to successful prevention of MTCT. The five-year incidence rate of probable and possible cases is 1 per 1000 live births (Table 1). The majority of cases (*n* = 7) were possible cases attributed to inadequate maternal treatment. We identified three cases of abortion and IUFD, probably due to untreated maternal syphilis infection (Table 2).

Latent syphilis was the primary diagnosis in most maternal cases (*n* = 9), with one case each of primary syphilis (Patient 6) and secondary syphilis (Patient 5). Treatment adequacy was a crucial factor in our sample, as several mothers received either inadequate treatment or no treatment prior to delivery. Among the neonates, two patients (Patient 1 and Patient 10) were identified as having intrauterine growth restriction (IUGR). Most infants were asymptomatic at birth with unremarkable physical examination.

The majority of cases did not present with multisystem involvement. Only one patient (Patient 11) presented with hepatosplenomegaly, which was further confirmed by abdominal computed tomography (CT) scan in addition to a finding of an isolated focal hepatic lesion. Laboratory findings including hematological changes were noted in two patients: one patient (Patient 11) was found to have cytopenia (anemia and thrombocytopenia) along with transaminitis and conjugated hyperbilirubinemia, while the second patient (Patient 2) was found to have anemia with reactive thrombocytosis.

Skeletal imaging identified abnormal long bone findings in two patients. The first patient (Patient 4) had cortical thickening of the bilateral shaft of both humeri as well as cortical irregularities along the shaft of the bilateral radius and ulna, suggestive of periostitis. The second patient (Patient 5) was found to have mild fraying and irregularity along the distal femoral metaphysis.

Regarding central nervous system involvement, all patients who underwent cranial ultrasound (*n* = 5) had unremarkable findings. Lumbar punctures were attempted in a few cases (*n* = 3); however, no cases of neurosyphilis were identified (all CSF VDRL results were negative).

The multisystem screening revealed no ocular involvement in any of the cases screened (*n* = 8); however, one patient (Patient 11) demonstrated abnormal audiological status upon hearing screening.

The majority of patients (*n* = 8) received a 10-day course of intravenous Penicillin G. Favorable outcomes were seen in patients who were followed up (*n* = 8), with all patients achieving normal neurodevelopmental outcome. The rest of the patients had lost their follow up.

One patient (Patient 4) was born to a mother with latent syphilis. The initial antenatal screening serology was performed at another center and was not available to us. The repeated syphilis screening during pregnancy at our center was rejected due to insufficient sample. During the third trimester of pregnancy, fetal ultrasound showed hepatosplenomegaly, mild ascites, short, long bones, and signs of fetal anemia requiring intrauterine transfusion. She was initially considered low risk for transmission of the infection, as she had completed three doses of benzathine penicillin 1 month prior to delivery. The infant presented at the age of 5 months with vomiting and failure to thrive, and the diagnosis of congenital syphilis was made based on the clinical picture and serology results. Maternal RPR at that time was 1:32 and TPHA > 1:2560.

Additionally, five infants (Patient 3, 7, 8, 9, and 10) were born to mothers with positive syphilis screening. The mothers were all asymptomatic and had positive initial serum screening (reactive or weakly reactive IgG and IgM against *Treponema pallidum*) with low RPR and TPHA. Although these cases were suspected to be false positives by the adult infectious disease team following these cases, the lack of follow-up to repeat the test during pregnancy could not rule out infection. Thus, the neonates born to these mothers were managed as possible cases of congenital syphilis. All the neonates were clinically asymptomatic, and all except one completed treatment with benzathine penicillin.

## 4. Discussion

In this paper, we describe the epidemiology of congenital syphilis at a single tertiary pediatric center in Riyadh, Saudi Arabia. We found the rate of congenital syphilis—calculated based on probable and possible cases—to be 1 per 1000 live births over the five-year study period.

In Saudi Arabia, data on congenital syphilis are limited. One study conducted at a university hospital in Riyadh screened 3270 pregnant women between October 2002 and March 2003 using RPR and confirmatory TPHA, reporting only a single case (0.03%) of maternal syphilis [15]. Another paper by Hossain et al. from 1987 reported an overall syphilis prevalence rate of 2.7% among 6684 screened sera. Among pregnant women, 4361 samples were collected and 37 were positive (0.85%). Paired maternal and neonatal sera were tested in 26 patients, and only one infant had a positive result [16]. This study was limited by the lack of confirmatory testing, which was not available at the time; therefore, may not reflect accurate rates of either maternal or congenital syphilis.

Global rates of syphilis infections in adults have been on the rise since 2018. This increase has also been reflected in the rates of congenital syphilis [2]. Locally, there is a scarcity of reported data on syphilis infection rates in adults. However, it is presumed that local syphilis infection rates in adults are low, as shown by syphilis serology screening of blood and transplant donors, reporting positivity rates ranging from 0% to 0.45% [17,18,19]. However, studies conducted in neighboring countries have shown higher prevalence. One paper published from a specialized STI clinic in the United Arab Emirates (UAE) reported a total of 290 cases between 2018 and 2022, 10% of which were maternal infections [20].

One systematic review reported abnormal antenatal sonographic findings in one third of the cases of congenital syphilis. The most frequent extra abdominal fundings were high middle cerebral artery peak systolic velocity, suggestive for fetal anemia (38%), placentomegaly (34%) and fetal growth restriction (14%), while abnormal CNS findings antenally are rarely reported. Intra-abdominal findings are most commonly due to hepatomegaly and splenomegaly (83% and 16%) [1]. In our cohort, prenatal antenatal ultrasound findings were abnormal in two out of twelve cases. One patient had multiple abnormalities involving the CNS, gastrointestinal and skeletal systems and the pregnancy ended in IUFD (Patient 1, Table 2). The other patient had IUGR (Patient 4, Table 1).

Postnatal image findings in our cohort were abnormal in 3/12 patients. Two had abnormal long bones and one patient had hepatomegaly and liver lesion. Consistent with other publications, hepatosplenomegaly, bone and skin involvement, are the most frequent findings in the post-natal ultrasound imaging [1].

Early treatment of maternal syphilis infection is associated with a significant decrease in the risk of transmission of the infection to the fetus. Specifically, starting penicillin G treatment prior to 28 gestational age was associated with congenital syphilis rates of 0.33 per 100 infants [21,22]. In our cohort, one patient developed congenital syphilis despite maternal treatment (Patient 4, Table 1). This incident is likely related to the delayed onset of therapy in the mother, due to delay in the diagnosis and engagement in care.

Saudi Arabia has implemented antenatal screening programs for early diagnosis and treatment of multiple sexually transmitted infections, including HIV, syphilis, and hepatitis B. This also aligns with the WHO triple elimination of mother-to-child transmission [5,6]. Historically, early screening coupled with engagement in care and nationwide immunization programs have led to excellent outcomes and zero vertical transmission rates of HIV and hepatitis B from mothers to their children [23,24]. Still, missed opportunities for early diagnosis have led to increases in the rates of congenital syphilis [25]. This is reflected in our cohort, as almost half of our probable congenital syphilis cases were attributed to delayed antenatal screening. Of these six cases, three resulted in abortion or IUFD.

Some limitations of this study are related to the missed follow-ups among some of the mothers with positive serology discovered during antenatal screening, who were not properly monitored after receiving treatment. This creates the possibility of overdiagnosis and overtreatment in mothers and neonates. Another limitation is the lack of syphilis screening in all cases of stillbirth and IUFD, which raises concern for underdiagnosis of congenital syphilis. Lastly, this study is limited by its single-center, tertiary-care design, which may restrict the generalizability of our findings. Larger, multicenter epidemiological studies across the region are urgently needed to better characterize the disease burden, identify key risk factors, and identify actionable opportunities to strengthen antenatal screening and prevention programs.

## 5. Conclusions

In this paper, we report congenital syphilis incidence rates that align with the global upward trend. We also identified multiple missed opportunities for screening and early treatment—findings that can inform future guidelines and enhance awareness among healthcare providers and the public. To address these gaps, we propose four actionable recommendations: (1) universal first-trimester screening with rapid confirmatory testing; (2) a centralized tracking systems for seropositive mothers to ensure post-treatment follow-up; (3) mandatory syphilis screening for all stillbirths and intrauterine fetal demises; and (4) enhanced provider education emphasizing the urgency of treatment initiation before 28 weeks’ gestation. Implementation of these interventions could substantially reduce congenital syphilis rates in our setting.

## Figures and Tables

**Table 1:** Summary of the cases of maternal syphilis infection and their respective neonates who were managed for congenital syphilis. IUGR = intrauterine growth restriction. RPR = rapid plasma reagin. TPHA = treponemal pallidum hemagglutination assay. VDRL = venereal disease research laboratory. CBC = complete blood count. LFT = liver function test. CSF = cerebrospinal fluid. IV = intravenous. * see text.

Case Number	Year of Diagnosis	Mother Diagnosis	Child	Diagnosis	Investigations
Patient 1. Male	2021	Late latent syphilis (2nd trimester) Inadequate treatment. (Received one dose) RPR 1:02 TPHA > 1:2560	Physical examination: unremarkable RPR 1:04 TPHA 1:160 IV Penicillin G for 10 days.	Possible	CBC and LFT: unremarkable. CSF: negative for VDRL. Audiological and Ophthalmological assessment: unremarkable.
Patient 2. Female	2022	Latent syphilis (2nd trimester) Adequate treatment. RPR 1:08 repeated 1:04 TPHA 1:160	Physical exam: unremarkable. RPR 1:02 TPHA 1:80 Didn’t receive treatment.	Less likely	CBC was remarkable for anemia, and LFT was unremarkable. CSF: not indicated. Audiological assessment: unremarkable. ophthalmological assessment: not done
Patient 3. Female	2022	Latent syphilis (2nd trimester). Untreated * RPR 1:01 TPHA < 1:80	Physical exam: unremarkable. RPR 1:01 TPHA < 1:80 Received IV Penicillin G for 10 days.	Possible	CBC and LFT: unremarkable CSF: not done. Audiological and ophthalmological assessment were unremarkable.
Patient 4. Female	2023	Latent syphilis (3rd trimester). Treated (see text). RPR and TPHA done in another center and not documented	IUGR (estimated fetal weight on the last scan at 4th centile, dropped from 7th, long bones < 5th centile) Physical exam: unremarkable. RPR 1:16 TPHA > 1:2560 Received IV Penicillin G	Highly Probable	CBC was unremarkable. CSF: not done Audiological and Ophthalmological assessment: unremarkable.
Patient 5. Female	2023	Secondary syphilis (2nd trimester). Inadequate treatment. RPR 1:04 TPHA 1:2560	Physical exam: unremarkable. RPR 1:02 TPHA 1:640 Received IV Penicillin G for 10 days.	Possible	CBC and LFT: unremarkable. CSF: not done (failed attempt). Ophthalmological assessment: unremarkable.
Patient 6. Female	2023	Primary syphilis (1st trimester) Received adequate treatment. RPR 1:16 TPHA 1:320	Physical exam: unremarkable. RPR: not available TPHA 1:160 Treatment not indicated	Less likely	CBC and LFT: unremarkable CSF: not indicated. Audiological and ophthalmological assessment: unremarkable.
Patient 7. Female	2023	Latent syphilis (3rd trimester) Inadequate treatment. RPR negative TPHA < 1:80	Physical exam upon delivery was unremarkable apart from requiring high flow nasal cannula. no end organ damage. Syphilis screening: equivocal. Received IV Penicillin G for 10 days	Possible	CBC and LFT: unremarkable CSF: negative for VDRL. Ophthalmological assessment: unremarkable.
Patient 8. Male	2023	Latent syphilis (3rd trimester) Inadequate treatment. RPR negative TPHA < 1:80	Physical exam: unremarkable. Syphilis screening: negative. Received IV Penicillin G for 10 days	Possible	CBC: unremarkable CSF: not done. Audiological and ophthalmological assessment: unremarkable.
Patient 9. Male	2024	Diagnosed as latent syphilis during pregnancy (2nd trimester) Didn’t receive adequate treatment. RPR 1:02 TPHA < 1:80	Physical exam: unremarkable. Syphilis screening: Negative. Didn’t receive treatment.	Possible	Initial workup including CBC was unremarkable LP was not done. Audiological assessment was unremarkable.
Patient 10. Female	2024	Diagnosed as latent syphilis during pregnancy (3rd trimester) Received 2 doses of penicillin. RPR 1:02 TPHA 1:160	Known case of IUGR (estimated fetal weight on the last scan at 4th centile) Physical exam upon delivery was unremarkable, no end organ damage. RPR 1:01 TPHA < 1:80 Received IV Penicillin G for 10 days	Possible	Initial workups including CBC and LFT test were unremarkable CSF negative for VDRL. Audiological and ophthalmological assessment were unremarkable.
Patient 11. Male	2024	Mother was not screened for syphilis during pregnancy. Serology after delivery: RPR 1:32 TPHA > 1:2560	Presented at 2 months of age with jaundice and hepatosplenomegaly. RPR > 1:128 TPHA > 1:2560 Received Ampicillin in another facility.	Highly Probable	Patient was found to have cytopenia (anemia and thrombocytopenia), he had conjugated hyperbilirubinemia. LP was not done for CSF VDRL. Ophthalmological assessment was unremarkable. He was found to have abnormal audiological screening.

**Table 2:** Stillbirth/abortions/IUFD secondary to probable congenital syphilis. IUFD = intrauterine fetal death. RPR = rapid plasma reagin. TPHA = treponemal pallidum hemagglutination assay. IM = intramuscular. GA = gestational age.

Case Number/ Year of Diagnosis	Mother	Comments
Case-1 2025	19-year-old, gravida 2 para1 + 0 Antenatal scan at 24 GA showed dilated lateral ventricle, small cerebellum, echogenic bowel, ascites and short femur and humerus. Underwent urgent amniocentesis. Delivered baby who is IUFD, at GA of 28 weeks +5	Syphilis screening done in the second trimester: TPHA > 1:2560 RPR > 1:128 Received 3 weekly doses of IM benzathine penicillin and one week of doxycycline.
Case-2 2025	38 years, Gravida 11 Para 5 + 5. Diagnosed with late latent syphilis in the premarital screen (second marriage). Current viable pregnancy, after the syphilis treatment was completed.	Syphilis screening done pre marriage: TPHA = 1:320 RPR = negative Received 3 weekly doses of IM benzathine penicillin.
Case-3 2022	41-year-old, gravida 6 para 4 + 1. Had spontaneous abortion at 7 weeks gestational age.	Antenatal syphilis screening: TPHA = 1:640 RPR = 1:04 Missed follow up (not treated).

## Data Availability

The data presented in this study are available on request from the corresponding author due to privacy and ethical related reasons.
